# Comprehensive Survey of Recent Drug Discovery Using Deep Learning

**DOI:** 10.3390/ijms22189983

**Published:** 2021-09-15

**Authors:** Jintae Kim, Sera Park, Dongbo Min, Wankyu Kim

**Affiliations:** 1 KaiPharm Co., Ltd., Seoul 03759, Korea; contact@kaipharm.com (J.K.); srpark@kaipharm.com (S.P.); 2 Computer Vision Lab, Department of Computer Science and Engineering, Ewha Womans University, Seoul 03760, Korea; 3 System Pharmacology Lab, Department of Life Sciences, Ewha Womans University, Seoul 03760, Korea

**Keywords:** artificial intelligence-based drug discovery, deep learning, drug–target interaction, virtual screening, de novo drug design, molecular representation, benchmark tool

## Abstract

Drug discovery based on artificial intelligence has been in the spotlight recently as it significantly reduces the time and cost required for developing novel drugs. With the advancement of deep learning (DL) technology and the growth of drug-related data, numerous deep-learning-based methodologies are emerging at all steps of drug development processes. In particular, pharmaceutical chemists have faced significant issues with regard to selecting and designing potential drugs for a target of interest to enter preclinical testing. The two major challenges are prediction of interactions between drugs and druggable targets and generation of novel molecular structures suitable for a target of interest. Therefore, we reviewed recent deep-learning applications in drug–target interaction (DTI) prediction and de novo drug design. In addition, we introduce a comprehensive summary of a variety of drug and protein representations, DL models, and commonly used benchmark datasets or tools for model training and testing. Finally, we present the remaining challenges for the promising future of DL-based DTI prediction and de novo drug design.

## 1. Introduction

The primary goal of drug discovery is to develop safe and effective medicines for human diseases. All the drug development processes—from target identification to step-by-step clinical trials—require significant amount of time and cost. As costs increase gradually with every step, it is essential to ensure that appropriate drug candidates are selected for the next phase at each milestone. In particular, the “hit-to-lead” process is a pivotal step in identifying promising lead compounds from hits and determining their potential as therapeutics. One of the reasons why clinical trials face side effects and lack in vivo efficacy is that single or multiple drugs often interact with multiple targets based on the concept of polypharmacology [1]. Ideally, full-scale in vivo tests for each disease model should be able to address this problem; however, that will require astronomical time and effort. Computer-aided drug discovery or design methods have played a major role in this hit-to-lead process by reducing the burden of consumptive validation experiments since the 1980s in modern pharmaceutical research and development (R & D) [2,3,4]. However, even this in silico approach has not prevented the decline in pharmaceutical industry R & D productivity since the mid-1990s.

Recently, much effort was invested in drug discovery through artificial intelligence (AI), which has enabled significant and cost-effective development strategies in academia and pharmaceutical industries. The vast amounts of chemical and biological data accumulated over decades, along with technological automation through the availability of high-performance processors such as graphics processing unit computing, paved the way for AI in drug development [4,5,6]. Not only state-of-the-art AI technologies are adopted in the drug development process, but also diverse pipelines or frameworks for AI-driven drug development are being built [7,8,9]. Utilizing deep neural networks provides the advantage of understanding the very complex contexts of biological space. This is because nonlinear models can be constructed in hidden layers to extract complex patterns from multi-level representations. It also minimizes the work of manually preprocessing unformatted raw data and selecting all kinds of features. Consequently, advances made in the development of deep learning (DL)-based methods have led to successful outcomes for prediction of drug–target interactions (DTIs) and generation of novel molecules with desired properties [4,10,11]. However, since datasets for drug development exhibit types and distributions that are different from those used in traditional AI data, such as images and texts, further attempts are still required to analyze data from a different angle and apply the latest DL techniques.

In this review, we introduce essential data representations and DL models for DTI prediction and de novo drug design. In addition, we investigate recent advances and benchmark datasets in DL-based methods in the following sections: The “Data Representation” section introduces several data representations of the inputs that were used in DL-based drug discovery. The “Deep Learning Models” section explains DL methods for drug discovery via comparison of the strengths and weaknesses of models. In the two sections, “Deep Learning Methods for Drug–Target Interaction Prediction” and “Deep Learning Methods for De Novo Drug Design,” we classify and describe the models for each of the DTI predictive models and de novo drug design models based on their utility. The “Benchmarking Datasets and Tools” section demonstrates the commonly used benchmark dataset and publicly available benchmarking tools. Finally, we discuss the advantages and limitations of the current methods as well as the remaining important challenges and future perspective for DL-based drug discovery in the “Limitation and Future Work” section. 

In the “Deep Learning Methods for Drug–Target Interaction Prediction” section, the description of the following studies was minimized: (1) studies that predict compound properties not considering protein targets such as blood–brain barrier permeability, solubility, lipophilicity, and chemical-based adverse effect [10,11]; (2) target prediction studies that determine targets for the existing drugs, such as reverse docking simulation [12]; (3) studies that only use knowledge-based documents from which information is extracted by text mining techniques [13]; (4) studies that focus on only optimizing binding between the drug and target using molecular dynamics simulation [14,15,16].

## 2. Data Representation

**Figure 1:**
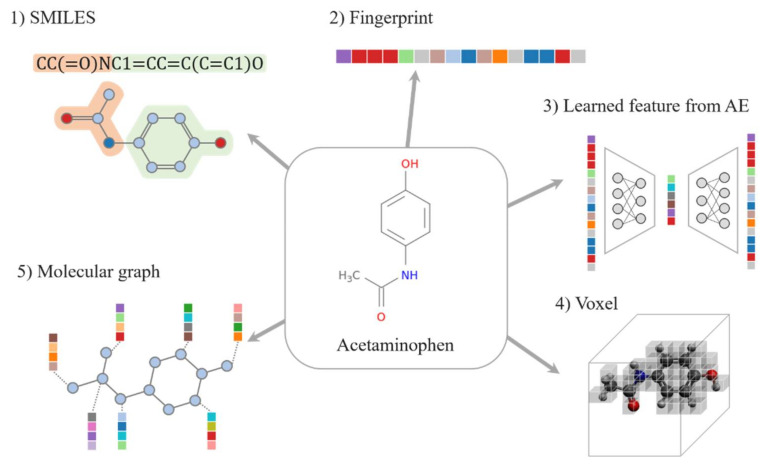
Different types of drug representations used in DL-based drug discovery. This figure shows the drug representations of acetaminophen, which is widely used to treat mild to moderate pain. (**1**) SMILES: a string that expresses structural features including phenol group and amide group. (**2**) Fingerprint: a 16-digit color-coded 64-bit MACCS key fingerprint. (**3**) Learned representations: In this case, it depicts the features learned from an autoencoder (AE). (**4**) Voxel: binary volume elements with atoms assigned to a cube with a fixed grid size. (**5**) Molecular graph: Each node encodes the network information of the molecular graph.

The input data for DL-based drug discovery are molecules; drugs and protein targets are small molecules or macromolecules. To characterize these molecules, several types of molecular representations (often referred to as descriptors or features) have been used in many machine learning (ML) methods—from simple sequences of molecular entities to manually predefined molecular features [17,18] (Figure 1). However, because it is directly related to the knowledge of learning models, data representation has a significant impact on pre-training to improve performance of predictive models. There has been a surge of interest in research on representation of molecules, and these efforts can contribute to capturing unknown features of compounds and targets [19,20]. Learning expressive representation from molecular structures is one of the challenges of these studies [21]. Besides, many recent DL models tend to use three-dimensional (3D) representations based on protein–ligand complexes, including molecular graphs, atom-pair fingerprints, and voxels. The various molecular representations utilized in deep neural networks as drug representations and target representations are described separately in this section.

### 2.1. Drug Representations

#### 2.1.1. SMILES

The most commonly used drug representation is a simplified molecular-input line-entry system (SMILES) string, which is a line notation that encodes structural, geometric, and topological properties of a molecule. The SMILES is simple and easy to obtain; therefore, it enables fast training. A number of molecular deep-learning models use the SMILES as the molecular representation [22,23,24,25]. As it is a sequence-based feature, a SMILES string can be directly used as a “sentence” to learn the representations. Sequence models using the SMILES can be successfully applied to predict chemical reactions. For example, many studies have shown promising results for synthetic prediction by directly converting to the predicted reactants in the SMILES format through seq-2-seq approaches to illustrate the reactions of compounds [26,27,28]. Furthermore, most molecules can be randomly generated with more than one SMILES string by starting in different atoms and changing the atomic orders, and this randomized SMILES model exhibits much better performance [29,30].

#### 2.1.2. Fingerprint

Another chemical structure-implemented feature is molecular FP, which is a bit string encoding structural or pharmacological feature of a ligand. Many types of molecular FPs have been proposed for similarity comparisons for virtual screening (VS), including ligand-based similarity searching and quantitative structure-–activity relationship (QSAR) analysis. A number of deep-learning-based DTI prediction models also used FPs as input features [24,31,32,33,34]. We discuss three types of widely used molecular FPs: key-based FPs, hashed FPs, and pharmacophore FPs.

Key-based FPs include molecular ACCess system (MACCS) and PubChem FP. The MACCS keys are composed of predefined 166 substructures. PubChem FP [35] has 881 bits and each bit tests the presence of element count, type of ring, atom pairing, and nearest neighbors, etc. These structural keys are designed for substructure retrieval. Therefore, although it is possible to quickly and accurately find substructures, there is a limit to classifying various characteristics.

Hashed FPs, such as Daylight FP, Morgan FP, extended-connectivity FP (ECFP), and functional-class FP (FCFP), are also used in the similarity analysis of compounds. Unlike key-based FPs, hashed FPs do not require predefined substructures and are instead created by a hash function to convert all possible fragments to numeric values. ECFP, a circular FP based on the Morgan algorithm [36], is often used in a wide range of applications, including DL models for the DTI prediction. This is because several DL methods using ECFP exhibited robustness in bioactivity prediction [8,37]. 

Pharmacophore FPs have pharmacophoric features such as aromatic, hydrophobic, charged, and hydrogen bond donor/acceptor. The pharmacophore FPs consider the overlapping of energy-minimized conformations of a set of known ligands and the extraction of recurrent pharmacophoric properties [38]. Many studies have used these pharmacophoric features to assess similarities between binding sites [39].

Finally, recent studies have attempted to add 3D structures to FPs to accurately predict binding affinity [8,40,41]. Gao et al. [33] reassessed the predictive power of 2D and 3D FPs and concluded that 2D FPs are still competitive in prediction of toxicity, physicochemical properties, and ligand-based binding affinity; however, 3D structure-based models outperformed 2D-based counterparts in the protein–ligand binding prediction. In other words, 2D FPs are still competitive; therefore, considering the structural properties at the 2D and 3D levels together will yield better results.

#### 2.1.3. Learned Representations

“word2vec” is a very popular method in natural language processing for word embedding [42]. With word2vec, the meaning of the word is learned and reflected in the coordinates; therefore, an ML model can better characterize the words. The embedding method, which usually adds “(2)vec” to the end, is inspired by “word2vec” and treats a molecule or protein as a sentence or word of a natural language and converts it into a real vector. ProtVec [43] and Mol2vec [44] are representative representations, and there are other methods such as SPvec [40] and SMILES2vec [41]. When converted to the 2vec type, specific information such as atom (or amino acid) type or bonding relationship of the original data cannot be known without restoration. Therefore, it is not suitable for a de novo design and is mainly used for property prediction. It is also known that the 2vec type has better prediction accuracy than the SMILES or FP [32].

There are other learned representation methods that employ DL. Recently, many studies used deep representation learning to encode molecules. The most common learned representation method is AutoEncoder (AE) [45]. The AE extracts the potential characteristics that make the input data distinguishable and compress them into vectors of desired length, called as latent vectors. Currently, the transformer model is preferred over the AE [46]. However, to train representation learning, a large amount of data is required; however, a publicly available pre-trained transformer or AE model can also be used without making your own DL model [46]. X-Mol [46] or MolGNet [47] are well-known frameworks designed for this purpose. By fine-tuning these frameworks according to the purpose, they can be used in a variety of ways—from property prediction, DDI, DTI, de novo design, to molecule optimization.

Additionally, Denis et al. applied wave transform for efficient representation of sparse voxel data [48], and Ziyao et al. proposed HamNet [49] considering molecular conformation in their study.

#### 2.1.4. Voxel

A voxel is a combination of “volume” and “pixel,” which is a data representation that extends a 2D image into three dimensions. In the 3D space, a value is assigned to the geographic location where the atom exists, and the rest is filled with zeros. The value can be 1 to indicate the presence of only an atom, and it may be an encoded value corresponding to the type of atom or a quantum chemical property such as hydropathy or electric charge [50]. In addition, as mentioned in Section 2.2, a voxel is used as the expression of the target protein and has the advantage that it can express only the pocket that reacts with the ligand instead of the entire protein [51]. Because the voxel has specific 3D information, it is a very suitable expression for binding prediction.

Resolution is important when using voxel. For example, if the size of a voxel is 20 × 20 × 20, the number of features in the input data is 8k, so the size is large; however, most of the data are filled with zeros. If the resolution is lowered to reduce the size, the accuracy will decrease, and if the resolution is increased, the data size and training speed will increase significantly. There are also 3D mesh [52] or point cloud [53] for geometric 3D representation; however, the voxel is most widely used in the DTI field.

#### 2.1.5. Molecular Graph

A molecular graph is a mapping of atoms constituting a molecule to nodes and chemical bonds to edges. In molecular graphs, nodes are sometimes represented using symbols in the periodic table to indicate atom types, or using some kinds of functional groups or fragments [20]. The edge attributes can describe bond strength or bond resonances between two atoms, which is important training data that is expressed as the adjacent matrix in the graph convolution neural network model [11]. With the development of graph neural networks, recent DL-based works have adopted molecular graphs as drug or target representations for both DTI prediction models and novel molecular design models. Notably, molecular graphs can represent not only 2D structures but also 3D structures with the spatial information including atomic coordinates, bond angles, and chirality. However, since the arrangement of atoms in a three-dimensional space changes constantly, the space in a molecular graph is almost infinite. Some successful results using 3D graph representation were obtained for the DTI prediction by avoiding inefficient computations [54]; however, the 3D structure data of the protein–ligand complex is insufficient. Thus, the model can memorize the features of the training data extensively [55]. If you need a further explanation of molecular graphs, we recommend referring to Ref. [20], which provides a good review of molecular representations.

### 2.2. Target Representations

#### 2.2.1. Sequence-Based Feature

The simple and primary feature of targets is a protein sequence composed of a linear composition of amino acid residues, which is easy to obtain and serves as the input of the recurrent neural network (RNN). Amino acid sequences including protein primary structures have been frequently used as a target representation in the predictive models. Information on protein primary structures can also generate a variety of target properties, such as monopeptide/dipeptide/tripeptide composition (also called protein sequence composition description; PSC), sequence motif, and functional domain. Lin Zhu et al. [56] listed the types of protein features derived from the amino acid composition: amino acid composition, sequence order, etc. Some studies [34,57] considered a position specific scoring matrix (PSSM) as the target feature. The PSSM is derived from an ordered set of sequences that are presumed to be functionally related and serves as an important feature that is widely used in the prediction of DNA or RNA binding sites (e.g., PSI-BLAST [58]) [34].

#### 2.2.2. Structure-Based Feature

For known protein structures, as previously described in the drug presentation part, molecular descriptors such as atom-pair map, voxel, and molecular graph were often used for the target representation to determine structurally matching ligands or to design new ligands for the protein [17,40,59,60]. However, there are not many known protein structures. Genetically encoded amino acid sequences determine the remarkable diversity of the molecular functions performed by finely tuned 3D structures (i.e., tertiary structure) through protein folding. Accurately predicting the folding structure of proteins in a real biological system is important in biomedicine and pharmacology. Scientists directly analyzed the stereoscopic structure of proteins using methods such as X-ray crystallography, nuclear magnetic resonance spectroscopy, or cryo-electron microscopy to decipher the structures of more than 170,000 protein species; however, it took a very long time to analyze a single protein. For this reason, the tertiary structure of proteins has been determined using computational methods. Since there are many variables in protein folding, this challenge has not been addressed over fifty years in computational biology [61]. Recently, DeepMind developed AlphaFold and succeeded in accurately predicting the 3D structure of proteins from amino acid sequences [62]. As these prediction results are open to the public, it is expected that all scientists will be able to gain new insights and spur their discovery in drug development.

#### 2.2.3. Relationship-Based Feature

The key questions for druggable targets are how to extract important features of the drug-binding site and how to predict potential space. Many DL models have simply applied amino-sequence-based features; however, some studies have focused on a variety of aspects of target proteins. Some groups [59,63,64] utilize not only sequences but also pathway membership information such as gene ontology (GO) terms [60] and MSigDB pathways [65]. A number of studies [59,63,66,67,68] employed a protein–protein interaction (PPI) network, which is generated into network-based features by node2vec or AE. Other studies showed the transcriptome data, such as connectivity map (CMAP) [69] and Library of integrated network-based cellular signatures (LINCS)-L1000 database [70], can be utilized as target features for the DTI prediction models [63,71]. The transcriptional profile is an integrated result of many genetic processes; thus, the characteristic changes in the transcriptional profiles can denote the underlying mechanisms of diseases of interest [72].

## 3. Deep Learning Models

Basic DL models can be classified according to their purpose, loss function, learning method, and structure. When DL was initially applied for drug development, there were studies using only a single model; however, recently, there are very few cases where only a basic model is used. In most cases, two or more of the basic models introduced below are combined. There are many different types of DL models, but only very basic ones have been described in this section. We describe the strengths and weaknesses of each model and introduce the characteristics of the models from the point of view of drug discovery.

### 3.1. Multi-Layer Perceptron

Multi-layer perceptron (MLP) is the most common neural network structure and is also called the fully connected layer, linear layer, etc. MLP’s strengths lie in classification and regression. Usually, it is trained by finding the optimal parameters that can minimize the error between the predicted value and the correct answer for the input. Since it is a standard model that has been studied extensively, various techniques have been established and almost all DL frameworks basically provide it; therefore, it is easy to apply, and stable performance can be expected. Because of its wide versatility, various data such as FP, transcriptome [71], bioassay [73], and molecular properties can be used along with the compound structure. Chen et al. used the MLP with four hidden layers for the DTI prediction, FP was used for compound, and various information such as PseAAC, PsePSSM, NMBroto, and structure feature were combined with the target protein information [57].

### 3.2. Convolutional Neural Network

Convolutional neural networks (CNN) extract local features by calculating several adjacent features through the same computational filter. By stacking the CNNs in several layers, global features including local features can be extracted. The CNN is generally used when all the input data are single-modal-like image recognition. The convolution filter is the same regardless of the amount of the input data. Even if the amount of the input data is large, the number of calculations increases; however, the number of parameters of the DL model does not increase much. Therefore, it is efficient for training. It is also relatively robust against noise from the input data. Because the CNN is well suited to atomistic geometry [74], it is often used in combination with the voxel or image-type data. DEEPScreen [19] used a 2D image of the molecule, and RoseNet [15], AK-score [75], and DeepDrug3D [51] predicted the DTI by converting the protein and ligand into a voxel. Although it is not optimized for sequential expression methods such as SMILES or amino acids, the CNNs are sometimes used instead of the RNNs [76]. Both DeepConv-DTI [31] and transformer-CNN [77] used the CNNs for sequential input data to build QSAR models.

### 3.3. Graph Neural Network

Most of the data used in ML is expressed in the form of a vector that matches the Euclidean space. In the case of single-vector-type data or sequential data, models such as MLP, RNN, or transformer can be used; however, it is not appropriate to apply these models to data expressed in relational graphs such as social networks. A graph neural network is a model designed to learn graph-type data in a DL method [78]. There are various graph types of data in drug discovery—compound structure, DTI relationship, PPI, patient–disease relationship, etc. There are several types of graph neural networks (GNN); however, the graph convolution network (GCN) [79,80] adopting the CNN method and the graph attention network(GAT) applying the attention mechanism are representative [47].

GNNs are widely used in many ways; however, they stand out primarily in three applications. The first is to predict the properties of compounds using representation learning. Yang et al. claimed that the GNN method that they employed has better property prediction performance than the existing methods [21]. According to this trend, recently, the GNN is widely applied for property prediction [81]. The second is to learn relationship information between different domains such as heterogeneous and bipartite [82,83]. For example, it is possible to learn the relationship between patients and diseases, and between genes and drugs, making it possible to utilize comprehensive meta data [83]. Lastly, in the field of de novo design, compounds are generated or optimized by the GNN [84].

### 3.4. Recurrent Neural Network

If sequential data are put into the recurrent neural network (RNN) one by one in order, it is influenced by the previous input value to derive the next output value. When the RNN was first introduced, AI performance in the natural language field, which was difficult to achieve previously, was greatly improved, and it became one of the famous models of DL. In addition, it can be used to embed structural information as a kind of representation learning by extracting the weight of the hidden layer and treating it as a feature with sequence information. However, the naive RNN has a simple structure, and there are performance limitations for application in various situations. The most important problem is the vanishing gradient problem, which exhibits poor performance for long-length data such as large proteins and large compounds because the length of the input sequence exponentially reduces the impact of items far from the currently entered item [85]. Moreover, since the same operation is executed repeatedly as the length of the input sequence, the length of the sequence increases the training time. Even when the items in sequential data have complex intrinsic relationships, their characteristics are not well learned.

Long short-term memory (LSTM) was invented in the 1990s and began to be widely used in the late 2000s [86]. LSTM was introduced to address the fast-vanishing problem of naive RNNs. The LSTM can be used with good performance even on longer sequential data compared to the RNN. Since its introduction, various modifications of the LSTM have been proposed [87], and recently, gated recurrent units (GRU) with a simpler internal structure [88] has also been widely used. It can simply be used for the de novo drug design, which randomly generates short-length compounds, and can generate an appropriate candidate drug by inputting a target protein sequence [89]. The LSTM and GRU have exhibited significant improvements over the RNN and are widely used to replace the RNN in drug discovery [31,90,91]; however, the vanishing problem still persists, which makes it difficult to use very long sequence data.

### 3.5. Attention-Based Model

The self-attention technique is a method that was first proposed by the transformer model to introduce machine translation in the field of natural language processing. Self-attention is a technique that calculates the association between the elements included in a sequence and extracts features for each element based on the calculated result. Unlike the RNN, which uses a single hidden state in which all time step values are implied, the attention technique handles past data in parallel; therefore, the correlation with distant tokens can be used without reduction. Furthermore, bidirectional encoder representations from transformers (BERT), introduced by Devlin et al. [92] in 2018, has dramatically improved natural language presentation using DL and has been actively introduced in drug discovery.

In DTI, the transformer model was naturally absorbed into the traditional QSAR modeling using the RNN. Karpov et al. [77] used a model applying CNN to a transformer with SMILES as an input to predict drug activity. Shin et al. [23] proposed a molecular transformer DTI (MT-DTI) model that predicts drug-target binding affinity by embedding the protein sequence using the CNN and embedding the molecule structure using the BERT. Lennox et al. [93] also proposed a model with a concept similar to MT-DTI; however, it used BERT for both protein and chemical structures and was based on GCN. Lennox et al. evaluated that their model performed better in predicting binding affinity than MT-DTI.

### 3.6. Generative Adversarial Network

Generative adversarial network (GAN), first published in 2014 [94], is the most representative generative model in the field of DL. The GAN is only used in the de novo drug design and not in the DTI. Two DL modules, generator and discriminator, are included in pairs, and these two modules are trained adversarially with each other, and finally, the generator produces fake results that cannot be distinguished from the real ones by a discriminator. Although the GAN is used as a very powerful method for some data types such as image data, it has difficulty in generating large molecules compared to other generative models. In addition, the technical difficulty for training is somewhat higher than that of other models, and it involves problems such as mode collapse.

The GAN, combined with reinforcement learning (RL), is considered a very successful model for novel molecules generation. There are various GAN architecture applications used in drug discovery [95], however, we introduce only two very simple models. Objective-reinforced generative adversarial networks (ORGAN) [90], introduced by Guimaraes et al., and molecular GAN (MolGAN) [91], introduced by Cao and Kipf, are frequently cited successful models. Since ORGAN uses SMILES data as input, sequence GAN (seqGAN) [96] is used as the basic framework and RL is added. ORGAN showed good performance in drug likeliness and synthesizability except solubility compared to the naive RNN. The MolGAN is a similar concept to the ORGAN, but it applied the GCN based on molecular graph representation and showed better performance than the ORGAN and the naive RNN.

### 3.7. Autoencoder

AE is a DL structure for basic unsupervised learning that consists of an encoder that compresses data and a decoder that reconstructs the data to their original shape. In this symmetric process, the dominant characteristic that distinguishes the data from each other is automatically extracted. A set of abstracted points compressed by the encoder, called a latent space, can be used in other models as new features. At the training stage, the encoder and decoder are trained simultaneously; however, after the training stage, only the encoder is separated and used for data embedding, dimension reduction, and visualization, or only the decoder is separated and used as a generation model. Because dimension reduction is possible without the need for data labels, it is good to use in combination with other DL models [97].

The points in the latent space created by the AE are very sparsely distributed; however, there is no continuous meaning between the points. Variational autoencoder (VAE) limits the latent space to a Gaussian-shaped stochastic fence. This increases the density of the latent space and makes the data continuous and smooth. Gómez-Bombarelli et al. showed that continuous search from one compound to another is possible in a smooth chemical latent space constructed using the SMILES [98]. AE is excellent in data compression and is used for the DTI, whereas VAE has lower compression performance than AE and is used for the de novo drug design due to the continuous and limited latent space characteristics described above.

Adversarial AE (AAE) [99] is a DL model that adds the GAN structure to the VAE, whose purpose is feature compression and generation. The VAE can compress the properties of compounds well; however, it exhibits inadequate performance to generate valid results. Conversely, the GAN can produce valid compounds and produce plausible results but can be biased with a single mode and have low diversity scores. Insilico Medicine first published the AAE [100,101] for the identification and generation of new compounds in 2016 [101], followed by an improved model named druGAN [102] in 2017. The AAE is a method that can show good performance in the generation of new compounds while compressing the data to the latent space. Polykovskiy et al. generated new compounds by changing the lipophilicity (logP) and synthetic accessibility of the input compound by adding the function to control the condition to AAE [103].

## 4. Deep Learning Methods for Drug–Target Interaction Prediction

DTI prediction using DL techniques incorporates both the chemical space of the compound and the genome space of the target protein into a pharmacological space, which is called as a chemogenomic (or proteochemometric, PCM) approach. This approach would ideally solve the DTI problem by building a chemogenomic matrix between the whole compounds and their biological proteins. Advances in the high-throughput screening (HTS) technology have enabled hundreds of thousands of compounds to be tested on biological targets in a very short time; however, it is practically impossible to obtain a complete chemogenomic matrix for a vast chemical space of 10^60^.

**Figure 2:**
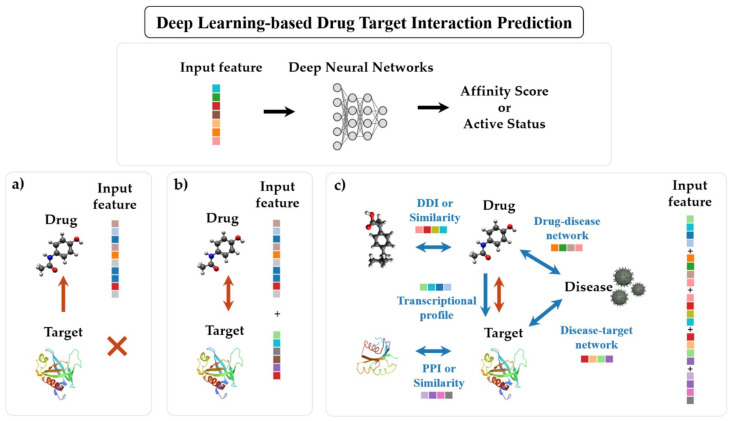
Deep learning-based drug–target interaction prediction. DL-based DTI prediction methods can be grouped based on their input features into three branches: (**a**) ligand-based approach, (**b**) structure-based approach, (**c**) relationship-based approach.

As DL models predicting DTIs continue to apply state-of-the-art algorithms, combine multiple algorithms, and gradually shorten development periods, DL-based DTI prediction models have become so diverse that it is difficult to divide groups into appropriate categories [104]. Previous review papers have categorized the DTI prediction models into various groups [104,105,106,107]; however, none of these reviews have established a clearly distinguished classification scheme for DL methods. Another review categorized ML-based DTI prediction methods into docking simulation methods, ligand-based methods, GO-based methods and so on; however, this also did not provide detailed classification of the DL methods [108]. Therefore, we summarized the recent works using deep neural networks as prediction models for the DTIs. We describe the works by grouping them into three branches according to their input features: (1) ligand-based approach, (2) structure-based approach, (3) relationship-based approach (Figure 2). Appendix A Table A1, Table A2 and Table A3 summarize the studies involving each approach.

### 4.1. Ligand-Based Approach

The ligand-based approach is based on the hypothesis that a candidate ligand will be similar to the known ligands of the target proteins. It predicts the DTI via the ligand information of the target of interest. This approach includes similarity search methods that follow the assumption that structurally similar compounds usually have similar biological activities [6,109,110]. For decades, these VS methods have either prioritized compounds in large compound libraries through tremendous computing tasks or solved problems using manual formulas. The DL technology can shorten these cumbersome steps and manual tasks, and the difference between in silico prediction and empirical investigation is gradually narrowed through deep neural network models (Appendix A Table A1). Researchers have developed deep-learning-based VS for exploring compounds with desired characteristics, which has led to the revival of new drug designs, which will be detailed in the “De Novo Drug Designs” section. 

With the development of benchmark packages such as MoleculeNet [111] and DeepChem [112], researchers can easily apply deep neural networks for analyzing ligands and predicting ligand-related properties, including bioactivities and physicochemical properties. Therefore, a number of ligand-based DL methods have adopted simple neural networks such as MLP and CNN [12,21,27,113]. In particular, ADMET studies tended to focus more on the representation power of the molecular descriptors than the model itself [27,34,35,114]. Hirohara et al. applied the SMILES string to a CNN model and detected motifs with important structures for protein-binding sites or unknown functional groups from learned features [25]. Wenzel et al. investigated multi-task deep neural networks using atom pairs and pharmacophoric donor–acceptor pairs as descriptors for predicting microsomal metabolic liability [115]. Gao et al. employed several ML algorithms, including random forest, single-task deep neural network, and multi-task deep neural network models, in order to conduct comparisons of six types of 2D FPs in the protein–ligand binding affinity prediction [33]. Matsuzaka and Uesawa developed a CNN model that predicts agonists for constitutive androstane receptors by training 2D images of 3D chemical structures [109]. They optimized the best performance in snapshots at different angles or coordinates of a 3D ball-and-stick model, and as a result, the approach outperformed the predictions of typical 3D chemical structures.

Several studies applied state-of-art techniques such as graph convolution network and graph attention network for bioactivity or physicochemical property prediction. Since the introduction of the GCN was introduced, GCN models in drug-related applications constructed graph representations of a molecule that included information about the chemical substructures by summing up all the features of all the adjacent atoms [116]. Many studies have applied the GCNs as 3D descriptors instead of SMILES strings and evaluated that these learned descriptors outperformed in the prediction tasks and are more interpretable than the existing descriptors [23,24,86]. Chemi-net utilized the GCN models for molecular representation and compared performances between single-task and multi-task deep neural networks on their internal QSAR datasets [81]. Yang et al. proposed an advanced model, the directed message passing neural network (D-MPNN), by adopting a directed message-passing paradigm. They extensively compared their models on 19 public and 16 internal datasets and found that the D-MPNN models performed better or exhibited similar performance in most of the datasets [21]. They underperformed compared to traditional 3D descriptors in two datasets and were not robust when the dataset was small or extremely imbalanced. Then, another study group also practically used this D-MPNN model and successfully predicted an antibiotic, called halicin, which showed bactericidal efficacy in mice animal models [22]. This became the first case that led to antibiotic discovery by exploring a large-scale chemical space with DL methods that cannot be afforded by the current experimental approaches. 

Another promising recent approach is the applications of attention-based graph neural networks [79]. Because the edge features can vary the graph representations for a molecule, the edge weights can be jointly learned with the node features. Thus, Shang et al. proposed an edge attention-based multi-relational GCN [11]. They built a dictionary of attention weights for each edge (i.e., individual bonds in the molecule), and as this dictionary is shared across the entire molecule, the model becomes robust to various input sizes. Consequently, the model can efficiently learn pre-aligned features from inherent properties of the molecular graph, and they evaluated that the performance of this model is better than that of the random forest model in Tox21 and HIV benchmark datasets. Withnall et al. [21] introduced a further augmentation with an attention mechanism to the MPNN model, called attention message passing neural network (AMPNN), which takes the weighted summation in the message passing stage [117]. They also extended the D-MPNN model (the reference by Yang et al. mentioned in the previous paragraph [21]) by attention mechanism in the same way as the AMPNN and called it the edge memory neural network (EMNN). This model outperformed other models on the standardized missing data from the maximum unbiased validation (MUV) benchmark set, although it is computationally more consumptive than other models.

### 4.2. Structure-Based Approach

Contrary to the ligand-based VS, structure-based VS uses both protein targets and their ligand information. Typical molecular docking simulation methods aimed at estimating geometrically feasible binding of ligands and proteins of a known tertiary structure [110]. While many ML methods for the DTI prediction utilize a variety of structural descriptors of ligands and targets as input features, several reviews separated these ML methods from typical structure-based approaches in methodology classification and classified them as feature-based methods [2,117,118]. However, we believe that the recent studies incorporating DL of feature-based methods utilize the same method because the training is essentially performed with structural features [17,40,59,119,120]. Appendix A Table A2 shows the recent applications of structure-based DL methods for the DTI prediction.

One of the most commonly used DTI prediction methods in recent years is the use of 1D descriptors for drug and target [26,33,37,42,121]. As described in the previous representation section, drug and target can be expressed as sequences of atoms and amino acid residues, respectively, and the sequence-based descriptors have been preferred because DL models can be applied immediately without any tricky preprocessing of input features. DeepDTA proposed by Öztürk [24] applied only sequence information of the SMILES string and amino acid sequences to a CNN model and outperformed moderate ML methods such as KronRLS [122] and SimBoosts [123] on the Davis kinase binding affinity dataset [113] and KIBA dataset [114]. Wen et al. chose the common and simple features, such as ECFPs and protein sequence composition descriptors, and trained the features by a semi-supervised learning through deep belief-network [37]. This study suggested that in a problem where a very sparse set of total DTI pairs is used for the training, even a small dataset can be predicted more accurately by unsupervised pre-training. Another work called DeepConv-DTI constructed a deep convolution neural network model using only a type of RDKit Morgan FP and protein sequences [31]. They additionally captured local residue patterns of target protein sequences from the pooled convolution results, which can give high values to important protein regions such as actual binding sites.

A keystone of the structure-based regression model is the score function that ranks the binding potential of the protein–ligand 3D complexes and parametrizes the training data to predict the binding affinity values or binding pocket sites of the target proteins. AtomNet incorporated the 3D structural features of the protein–ligand complexes to the CNNs [124]. Their understanding is that the interactions between the chemical groups in the protein–ligand complexes are predominantly constrained in a local space; therefore, the CNN architecture is appropriate to learn local effects such as hydrogen bonding and π-bond stacking. They vectorized fixed-size 3D grids (i.e., voxel) over the protein–ligand complexes, and then each grid cell represents the structural features in that location. Since then, many researchers have investigated deep CNN models using voxels for binding affinity prediction or binding pocket site prediction [17,40,59,60,120], and these models have shown improved performance compared to the popular docking methods such as AutoDock Vina [125] or Smina [126]. This is because the CNN models are relatively resistant to noise in the input data and can be trained even when the input size is large.

Similar to the trends in the ligand-based methods, many DTI studies based on the structure-based methods using the GCNs have been published [8,127,128]. Feng et al. adopted both the ECFPs and GCNs as ligand features [8]. Compared to previous models such as KronRLS [122] and SimBoost [123], their models showed better performance on the Davis [113], Metz [129], and KIBA [114] benchmark datasets. However, they acknowledged that their GCN model could not beat their ECFP model because of difficulties in applying the GCN due to time and resource constraints. Another DTI prediction study by Torng et al. built an unsupervised graph-AE to learn the fixed-size representations of the protein-binding pockets [118]. Then, they used the initialized protein-pocket GCN in the pre-trained GCN model, while the ligand GCN model was trained using the automatically extracted features. They concluded that this model effectively captured the protein–ligand binding interactions without relying on the target–ligand complexes.

Attention-based DTI prediction methods have emerged because the attention mechanism-implemented models have key advantages that make the model interpretable [25,128,130]. Gao et al. used encoded vectors using the LSTM recurrent neural networks for the protein sequences and the GCN for ligand structures [119]. In particular, they focused on explaining the ability of their approach to provide biological insights to interpret the DTI predictions. To this end, two-way attention mechanisms were used to compute the interaction of the drug–target pairs (DTPs) interact, enabling scalable interpretability to incorporate high-level information from the target proteins, such as GO terms. The Molecule transformer DTI (MT-DTI) method was proposed by Shin et al. using the self-attention mechanism for drug representations [23]. The pre-trained parameters from the publicly available 97 million compounds (PubChem) were transferred to the MT-DTI model, and it was fine-tuned and evaluated using two Davis [113] and KIBA [114] benchmark datasets. However, they did not apply the attention mechanism to represent the protein targets because the target sequence length was long, which takes a considerable amount of time to calculate, and there is not enough target information to pre-train. On the other hand, AttentionDTA presented by Zhao et al. combines an attention mechanism for the CNN models to determine the weight relationships between the compound and protein sequences [120]. They demonstrated that the affinity prediction tasks by the MLP model performed well on these attention-based drug and protein representations.

### 4.3. Relationship-Based Approach

According to polypharmacology, most compounds have more effects not only on their primary targets, but also on other targets. These effects depend on the dose of the drug and the related biological networks. Therefore, in silico proteochemometric modeling turned out to be useful, particularly when profiling selectivity or promiscuity of the ligands for proteins [121]. Moreover, multi-task learning neural networks are well suited for learning aspects of these different types of data simultaneously [131]. There are many applications of DL models that utilize relational information for multiple perspectives such as DTI-related heterogeneous networks and drug-induced gene-expression profiles. A network-based approach uses heterogeneous networks that integrate more than two types of nodes (drugs, target proteins/genes, diseases, or side effects) and various types of edges (similarities between drugs, similarities between proteins, drug–drug interactions (DDIs), PPIs, drug-disease association, protein-disease association, etc.) [132,133]. 

The key point of this approach is the use of local similarity between the nodes in the networks. For example, when a similarity network with drugs as nodes and drug–drug similarity values as the weights of the edges is considered, the DTIs can be predicted by utilizing their relationships and topological properties. It is based on the “guilt-by-association” theory that interacting entities are more likely to share functionalities [13]. Various ML methods that incorporate heterogeneous networks have been used as the prediction frameworks, e.g., support vector machine [134,135], regularized least square model (RLS) [127,128,136], and random walk with the restart algorithm [122,137].

With growing interest in the use of DL technologies, network-based DTI prediction studies using DL have been shown to improve the existing association prediction methods for measuring the topological similarities of bipartite (drug and target networks) and tripartite linked networks (drug, target, and disease networks) [15,69,73,74,104]. Zong et al. exploited the tripartite networks through the application of the DeepWalk method [130] to obtain the local latent information and compute topology-based similarities, and they demonstrated the potential of this method as a drug repurposing solution [13]. 

Some network-based DTI prediction studies used relationship-based features that were extracted by training the AE. A DTI-CNN prediction model devised by Zhao et al., used low-dimensional but rich depth features in a heterogeneous network trained by the stacked AE algorithm [68]. Moreover, in vivo experimental validation on atherosclerosis found that tetramethylpyrazine could attenuate atherosclerosis by inhibiting signal transductions in platelets. Other two studies [59,97] also applied the AE to capture the global structure information of the similarity measures. A study by Wang et al. applied a deep AE and introduced positive pointwise mutual information to compute the topological similarity matrix of drug and target [59]. Meanwhile, another study by Peng et al. utilized a denoising AE to select network-based features and reduce the dimensions of representations [97]. The denoising AE adds noise to high-dimensional, noisy, and incomplete input data and enables the encoder to learn more robustly by making the self-encoder learn to denoise.

However, these approaches have a limitation in that it is difficult to predict new drugs or targets, which is well known as the “cold start” problem of the recommendation systems [138]. These models are strongly influenced by the size and shape of the network; thus, if the network is not sufficiently comprehensive, they do not capture the properties of all the drugs or targets that may not appear in the network [13,139]. 

The other approach is using transcriptome data for DTI predictions, which measures the biological effect of drug action in in vitro experimental conditions. After the first release of the CMAP [69], a large-scale drug-induced transcriptome dataset, there have been many studies that succeeded in the identification of the drug repositioning candidates for a variety of diseases or the elucidation of the drug mode of action [140,141,142,143]. A number of studies have also employed the gene-expression profiles as the chemogenomic features for predicting DTIs. These studies are based on the assumption that drugs with similar expression profiles affect common targets [144,145].

Recent studies incorporated the updated version of CMAP, LINCS-L1000 database [70] into the DL DTI models [67,77,146]. Xie et al. built a binary classification model using a deep neural network based on the LINCS drug perturbation and gene knockout results [71]. On the other hand, Lee and Kim used the expression signature genes as the input drug and target features. They trained the rich information considering three distinct aspects of protein function, which included pathway-level memberships and PPI extracted using node2vec [63]. DTIGCCN by Saho and Zhang used a GCN model to extract the features of drug and target, respectively, from the LINCS data and CNN model to extract the latent features to predict DTPs [147]. In this hybrid model, they found that the Gaussian kernel function was helpful in building high-quality graphs; thus, their model showed better performance on classification tasks. The relationship-based DL methods described above are provided in Appendix A Table A3.

## 5. Deep Learning Methods for De Novo Drug Design

**Figure 3:**
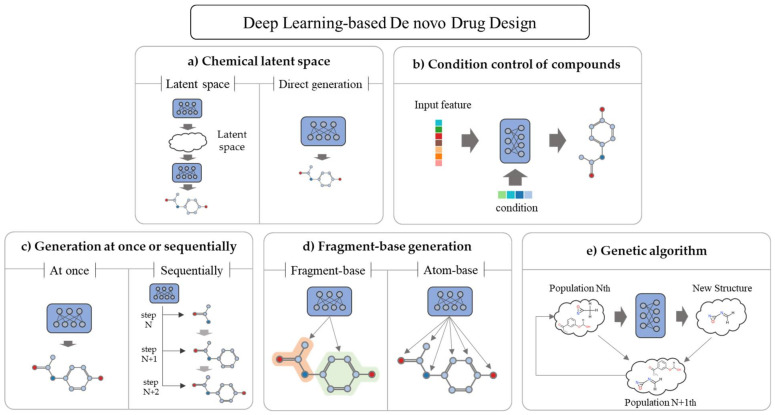
Deep learning-based de novo drug design. DL-based de novo drug design can be classified into five types according to function and method. (**a**) Classification according to the presence or absence of chemical latent space using manifold learning. (**b**) Classification according to the existence of condition control function. (**c**) Classification based on sequential generation. (**d**) Classification based on whether the molecule is produced in fragments or atoms. (**e**) Classification according to genetic algorithm using DL.

In general, when classifying the de novo drug designs, studies are classified based on the DL models [87]. However, in the case of actual implementation, it may be an appropriate classification; however, it may not be enough to understand the purpose of the model. In reflection of the recent trend changes, purpose, and usability, drug design using DL has been newly classified (Figure 3).

### 5.1. Chemical Latent Space

The manifold hypothesis states that there exists a low-dimensional subspace that explains specific data well within the original data space [148]. The latent space is mapped with a relatively low-dimensional vector space. The latent space created through manifold learning expresses the potential characteristics of the input data well. A representative model that performs manifold learning is the AE. Dimension reduction methods based on mathematical algorithms such as principal component analysis (PCA), t-distributed stochastic neighbor embedding (t-SNE), and singular value decomposition (SVD) have similar functions; however, they have limitations in determining complex manifolds compared to the ML methods.

Converting to a latent vector as an input feature has several advantages. First, the dimension of input data is reduced. Reducing the dimension reduces the risk of overfitting the module and makes learning easier with less data. Second, it becomes possible to search or optimize molecules in the latent space [149,150,151,152]. Since similar compounds or proteins are more densely expressed in a well-trained latent space [153,154], it is also possible to compare properties or calculate compounds according to compound structures [98]. A model named GENTRL proposed by Zhavoronkov et al. [7] generated compounds reflecting the latest trends that are indirectly extracted from patent dates using self-organizing map (SOM) from the chemical latent space. Instead of learning the encoder separately, if it is connected to a DL model and learned simultaneously during the training process, a latent space is produced that is more suitable for the purpose.

However, since the latent space is also data-dependent, it is produced differently each time, and it may be constructed differently or in an incomprehensible form unlike human intention. Recently, pre-trained encoders such as X-MOL [46] are provided as independent modules. By using transfer learning, described in Section 7.2, the performance can be improved by fine-tuning the latent space trained for a universal situation according to an appropriate purpose.

### 5.2. Condition Control of Compounds

There are two methods for studying new drug candidates in the de novo drug design using DL. The first method is generating as many arbitrary compounds as possible and filtering them through several steps according to the purpose and finally determining a small number of candidate drugs [7]. The second method is forcing conditions or properties to meet the purpose of the generation [45]. It cannot be said that either one is better; however, many random generation methods were used in the relatively early days, and recently there are many studies on controlling the condition. Condition-controllable models are useful when creating new drugs or optimizing the existing drugs because the condition control model can modify properties such as binding affinity, logP, molecular weight, side effects, and toxicity while maintaining the main structural characteristics of the molecule.

**Figure 4:**
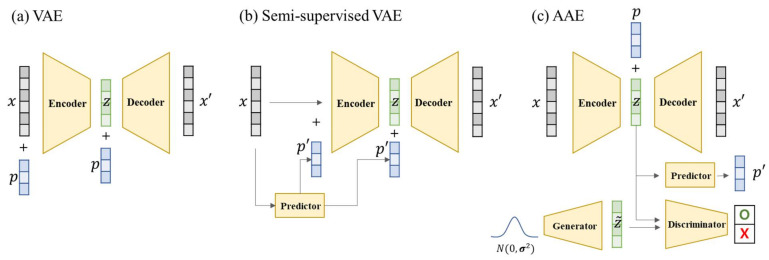
Three types of conditional de novo model using VAE. z is a latent vector and p is a molecular property. (**a**) Basic model for property control [45]. Concatenate the molecular property with the input value to the encoder and decoder. (**b**) Model with the property predictor [155] added to (**a**). It is possible to train even for molecules without the property data. (**c**) Model with condition control applied to the AAE [156]. Modify the latent space by predicting properties from the latent vector. This model does not add any properties to the encoder.

**Figure 5:**
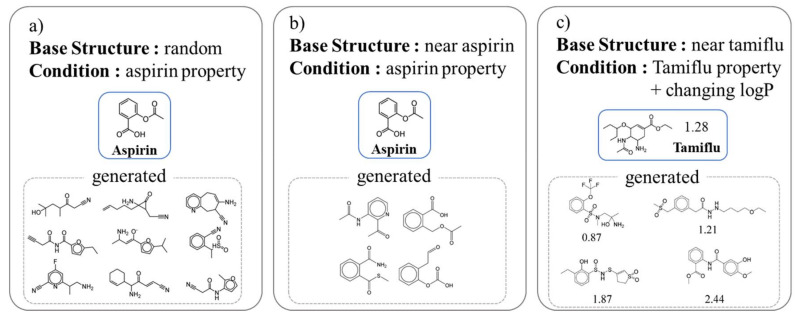
Generated compounds from conditional VAE. (**a**) Compounds created by adding aspirin condition to random points (compound structure) in the latent space. Although the form is different, they have similar properties (MW, logP, HBD, HBA, and TPSA). (**b**) Compounds produced by controlling the properties of aspirin at random points close to aspirin in the latent space. It looks very similar to aspirin. (**c**) Compounds produced by changing log P while maintaining the structure and other properties of Tamiflu. Only the desired properties in the reference compound can be controlled and improved. This figure is modified from [45].

There are various locations and methods of applying the condition (Figure 4). Lim et al. [45] manipulated the properties of the compound to be produced using conditions in the VAE (Figure 5). This simple model is trained by adding molecular properties (MW, logP, HBD, HBA, topological polar surface area (TPSA)) to both the encoder and decoder of the VAE. When creating a new compound, the researcher only needs to add the desired property to the latent vector that determines the structure of the compound. The difference between the input value and the output value is approximately 10%. Lim et al. conducted an interesting experiment to observe the change in the condition. The first was to create a set of compounds with similar properties but different structures by putting the properties of aspirin in a random latent vector. Second, several compounds similar to aspirin were generated by adding the properties of aspirin to a latent vector near aspirin. Finally, similar to the second experiment, the properties of Tamiflu were added to the latent vector near Tamiflu; however, the structural change was observed while changing the logP to various values. Kang et al. [155] made a model almost similar to Lim et al.’s model [45]. However, while Lim et al.’s model can use only compounds with known properties, Kang et al.’s semi-supervised VAE (SSVAE) can use more compounds for training by inputting the predicted results from the property predictor. Hong et al. [156] suggested different structures in their two previous studies. They used the AAE model instead of the VAE and connected the property predictor from the latent vector to refine the latent space, and then they put the compound properties (logP, SAS, and TPSA) together with the latent vector in the decoder in the training process.

### 5.3. Generation at Once or Sequentially

Basic generative models such as the AE or GAN form a compound from a corresponding input vector by a decoder or generator at once. In the case of the RNN using the SMILES, the word with the highest probability of matching the grammar is generated one by one from the start token until the end token appears, and finally, a large compound is completed. The one-time generation method is a method to create a new compound directly in the latent space, whereas the sequential method can start from nothing or a specific substructure and gradually complete the compound. The one-time method is simple and can provide more diverse results. Since the sequential method is generated while maintaining the active site or core scaffold with core characteristics, it can improve the binding score or properties; therefore, it can be used for fine lead optimization. Grisoni et al. [157] generated novel compounds using the SMILES and RNNs and Bongini et al. [158] using the GNNs. Lim et al. [84] created a graph-based sequential generative model from a specific scaffold rather than an atom, which has a property (MW, TPSA, logP) control function.

### 5.4. Fragment-Based Generation

**Figure 6:**
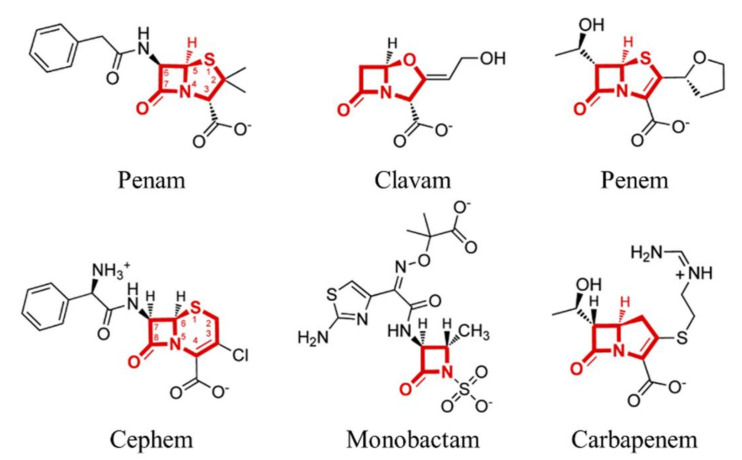
Chemical structures of the selected examples from six β-lactam structural categories. The core scaffolds (highlighted in red) are similar. Figure modified from [160].

A typical structure-based molecular representation, such as the SMILES or graph, consists of atoms and their junctions. However, compounds have more similar properties at the scaffold level than at the atomic level. The advantage of the fragment-based DL models is that when generating relatively large molecules, they output a product that is likely to exist in the natural state. For example, in the case of an atom-based model, the produced compound may include a ring consisting of 10 carbons, or a very long linear compound consisting of carbons, which are rare in nature. However, first, if the scaffold is used as a reference instead of the atom, it can be trained and created while maintaining the main substructure of the compound [84,146]. Second, it is easy to interpret and give feedback on the results based on the experts’ existing knowledge [159]. For example, beta-lactam has a characteristic scaffold (Figure 6) [160] so when developing a new antibiotic, various types of drugs can be created while maintaining the scaffold. Jin et al. [161] used molecular graphs to generate compounds from fragments (they called them motifs), and Arús-Pous et al. [146] used the SMILES to model adding fragments (they called them decorators) from a core scaffold.

Fragment-based generation has a limitation in that it becomes difficult to find a new molecular entity because only compounds similar to the existing scaffold structure are generated. In addition, there are very few types of atoms and bonds that are used in molecules, whereas the scaffolds have many types if not restricted by certain criteria.

### 5.5. Genetic Algorithm

Genetic algorithm is a method inspired by biogenetics, which has been traditionally used before DL [162], and it is mainly used to address optimization problems. The algorithm generates a random set of data called the initial generation and combines them to create a new generation. It repeats the process of intersecting some data with the highest score to create the next generation to obtain the most optimal result. If each data can be expressed in the form of a gene, and if there is a titration function that can be evaluated as a continuous value, it can be introduced relatively easily and produces a sufficiently acceptable result, although not perfect. De novo drug design using a genetic algorithm has been studied until recently [163,164], and the genetic algorithm method combined with DL [165] has been proposed in recent years.

## 6. Evaluation Method

### 6.1. Benchmarking Datasets and Tools

Over the decades, large amounts of repositories on the bioactivity, structure, and protein targets of small molecules have been accumulated in public databases such as PubChem [163], ChEMBL [164], and BindingDB [166]. These big datasets enable us to build predictive models for drug–target relationships via computational methods. To compare the performance of the models by evaluating their reproducibility for the prediction results, several datasets have been used. Appendix A Table A4 shows the list of benchmark datasets. These datasets consist of known active data and inactive compounds. In many datasets, an inactive compound is presumed to be inactive unless it is identified as active in an experimental biological assay, also referred to as “decoys” [167]. There are several benchmarking databases that provide refined decoy compounds [166,168]. They rationally selected inactive compounds to avoid false negatives because the false negative (i.e., active compounds are considered as inactive in the decoy sets) can underestimate the performance of the prediction methods. DUD-E [166], a gold standard dataset used for the evaluation of the VS methods, selected decoys based on the concept that the decoy compounds must be structurally different from the known ligands to reduce the false negative, whereas it must be similar to the known ligands with respect to physicochemical properties to reduce bias.

Chen et al. argued that the hidden bias in the widely used dataset (DUD-E database) may lead to misleading performance of the CNN models during the structure-based VS [169]. There were two remaining biases [170]. One is the limitation of exploring the decoy restricted in the chemical space of reference compounds including active compounds (i.e., analogous bias). The other is the limitation of artificially good enrichment in evaluation because the physicochemical properties of the active compounds and decoy compounds (i.e., artificial enrichment bias) can be clearly distinguished. To overcome these limitations, the MUV datasets [168] and the demanding evaluation kits for objective in silico screening (DEKOIS) [171] were proposed. Until recently, fine-tuned benchmarking datasets have been consistently presented. Xia et al. proposed the unbiased ligand set (ULS) and unbiased decoy set (UDS) [172] for the G protein-coupled receptors. Another group used an asymmetric validation embedding procedure to design a novel dataset called LIT-PCBA dataset [173] for some PubChem bioassays.

Candidate drugs created using the de novo drug design cannot be measured for efficacy unless they are actually synthesized. The de novo drug design using DL has developed significantly in recent years [19], and designing a good generative model rather than generating an effective drug is a major evaluation criterion in the de novo drug design research field. MOlecular SEtS (MOSES) [174] and GuacaMol [175] are the most popular benchmarking tools. These two tools score and compare the performance of new DL models based on the base models. Both the tools use post-processing databases based on ZINC or ChEMBL, and include general representations such as FP, SMILES, and molecular graphs. The features and issues of both the tools for benchmarking in the de novo drug design are described in detail by Grant et al. [176] in their review paper.

### 6.2. Evaluation Metrics for DTI Prediction

The DTI prediction can be grouped into two types: (1) DTP prediction by a classification model that assigns a positive or negative (i.e., active or inactive) label to the DTP and (2) drug–target affinity (DTA) prediction by a regression model that estimates the binding affinity value between the drug and target. Because the evaluation metrics are different for each type, this section describes the performance metrics for each type of model.

#### 6.2.1. Classification Metrics

The DTP prediction studies have adopted several common evaluation indicators including accuracy, precision, recall (also known as sensitivity), and specificity. These metrics are calculated from the confusion matrix. The most straightforward metric for classifier performance is accuracy. However, the accuracy metric does not work well in problems with skewness or class imbalance. For example, a prediction with a target that only affects less than 1% of all drugs is very easy to achieve 99% accuracy by obtaining the correct negatives even when few correct positives are predicted. For this reason, precision and recall are often quantified by many DL studies, which measure that the correctly predicted DTI with activity in practice is classified as positive repeatedly. Precision and recall can be measured simultaneously using two scores: F-score and precision–recall area under curve (AUPR). F-score (also called F measure) is a balance between precision and recall. For example, an F1 score is the weighted average value of precision and recall. The PR-AUC can show the tradeoff between precision and recall and reduce the impact of false positives. There are other useful metrics when the classes are imbalanced. Balanced accuracy applies the average of the sensitivity and specificity [177]. Matthews correlation coefficient (MCC) measures the correlation of the true classes with the predicted labels. Chicco and Jurman [178] showed that MCC is more informative in evaluating binary classifications than accuracy and F1 score.

Another commonly used evaluation indicator for DL methods is the area under the curve (AUC). The AUC means the area underneath a receiver–operator characteristic (ROC) curve that compares the performance of classifiers by distinguishing the two types of errors: false positives or negatives. The ROC curve is the plot of true positive rate (TPR, or sensitivity) against false positive rate (FPR, or 1-specificity), and the best classifier that will achieve perfection is the top-left of the plot (FPR = 0, and TPR = 1). The AUC value in the DTI prediction indicates how well the positive DTIs are ranked in the prediction. The AUC is sensitive to the imbalanced DTI dataset, which are prone to a large number of false positives (i.e., few positive DTIs relative to negative DTIs) [179].

#### 6.2.2. Regression Evaluation Metrics

Binding affinity scores such as IC50 and pKd predicted by DTA prediction models can be assessed by several evaluation indicators: mean square error (MSE), root mean square error (RMSE), Pearson’s correlation coefficient (R), and squared correlation coefficient ($R^{2}$). These metrics have been implicated to determine the quality of predictive QSAR models. The MSE is defined as the average squared difference between the predicted and ground-truth binding affinity scores. The RMSE, as its name suggests, is the squared RMSE. $R^{2}$ measures how well the predicted values match the real values (i.e., goodness of fit). Some studies [23,120] applied the modified $R^{2}$ ($r_{m}^{2}$) to the test set prediction, which was introduced by Roy and Roy [180].

Other metrics such as concordance index (CI or C-index) and Spearman’s correlation coefficient (ρ) quantify the quality of rankings by comparing the order of the predictions and the order of the ground truths. A frequently used ranking metric in the DTA prediction is the CI [25,130,181]. When predicting the binding affinity values of two random DTPs, the CI measures whether those values were predicted in the same order as their actual values. The other metric, Spearman’s correlation coefficient, measures the strength and direction of the association between two ranked variables. Several studies utilized Spearman’s correlation coefficient with other metrics [17,59,60,182].

### 6.3. Evaluation Metrics for De Novo Drug Design

#### 6.3.1. Generation Metrics

**Figure 7:**
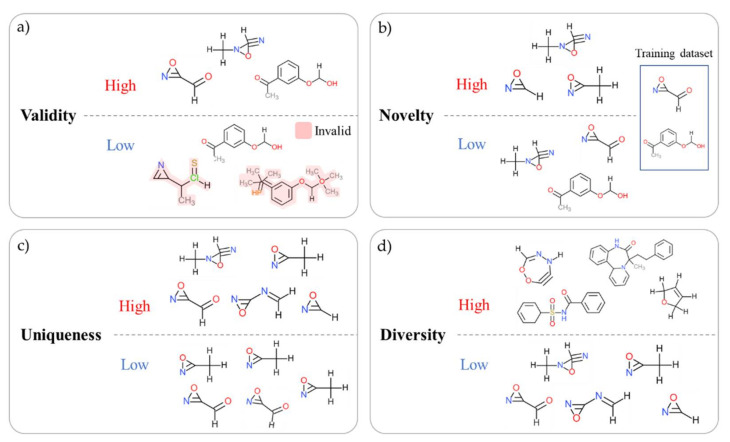
Generation metrics for de novo drug design. The four generation indices are commonly used in the de novo drug design studies: (**a**) validity, (**b**) novelty, (**c**) uniqueness, and (**d**) diversity. Unlike other metrics, a training dataset is required to measure novelty.

The generation index is meaningful in evaluating the performance of the DL generator model through the set of generated molecules rather than evaluating the generated compounds as drugs. This does not mean that models with better generative metrics make better drugs. The four generation indices are commonly used in the de novo drug design studies, and in the case of the SMILES data, an open library such as RDkit [119], GuacaMol [175], or MOSES [174] can be used to quickly measure the generation index (Figure 7).

Validity index evaluates whether a generated compound can exist or not. For example, in the case of the SMILES expression method, if the grammar is not learned sufficiently, the valid molecules are not generated, or the parentheses do not match. Validity is the ratio of compounds that grammatically exist among all the compounds, and the closer to 1, the better the model. A higher validity may indicate a better model; however, from an industrial point of view of new drug development, low validity is not necessarily a problem. Even a model with low validity can increase the absolute number of valid compounds by securing a large population. This is because the additional cost of generating more compounds and filtering valid compounds in the VS stage is relatively low compared to the time and cost in other stages. Rather, if the novelty and uniqueness performance is lowered to increase the validity, it may not be suitable for the creation of new drug candidates.

Uniqueness is a number that determines whether the generator creates a new compound without duplication. Compared to other types of data such as images and sounds, a compound is a very discrete type of data. For this reason, even if a small change or noise is added to the input condition, the generated compound does not reflect the change, and the same compound may be created repeatedly. Uniqueness is evaluated by the number of generated products and the ratio of unique compounds with duplicates removed. If the uniqueness is 1, it means that all the generated compounds are different without duplicates.

While uniqueness measures the absence of overlap within the generated compound set, novelty measures the non-overlapping property by comparing the generated set with the existing dataset. That is, it evaluates whether the generator has created a new compound that does not exist in the training dataset. It is evaluated by the ratio of the subset with the training dataset compared to the generated compound. The closer the novelty is to 1, the more completely new compounds are created, and although it is used as an important indicator in the field of de novo drug design, it is not important if the existing compounds are also allowed.

(1) $$\mathrm{Validity}\left(N\right)=\frac{\#\;of\;valid\;compounds\;in\;N}{\#\;of\;compounds\;in\;N},$$

(2) $$\mathrm{Uniqueness}\left(N\right)=\frac{\#\;of\;unique\;compounds\;in\;N}{\#\;of\;compounds\;in\;N},$$

(3) $$\mathrm{Novelty}\left(N,\;T\right)=\frac{\#\;of\;intersection\;between\;N\;and\;T^{c}}{\#\;of\;compounds\;in\;N}.$$

N = generated compounds set, T = Training dataset, $T^{c}$ = Complement set of T Diversity or dissimilarity (or distance) is an indicator to determine how dissimilar and diverse the produced compounds are when only a few scaffolds or a small number of atoms are changed. Chemotype diversity can be measured as a value between 0 and 1 using scaled Shannon entropy. Similarity can also be measured using the distance between compounds the expressed in the FP or SMILES. As shown in Equations (4)–(6), the average distance within a set can be calculated using the Tanimoto coefficient [183].

(4) $$\mathrm{Tan}\mathrm{imoto}\;\left(x,\;y\right)=\left(\frac{x\cdot \;y^{T}}{x\cdot \;x^{T}+y\cdot \;y^{T}-x\cdot \;y^{T}}\right),\;$$

(5) $$\mathrm{Soergel}\;\left(x,\;y\right)=1-\mathrm{Tan}\mathrm{imoto}\;\left(x,\;y\right),\;$$

(6) $$\mathrm{Distance}\;\left(N\right)=\frac{2}{N_{u}^{2}}\;\sum_{i=1}^{N_{u}-1}\sum_{j=i+1}^{N_{u}}\mathrm{Soergel}\;\left(x_{i}^{u},x_{j}^{u}\right).\;$$

Controllability is mainly used in the de novo models with the condition control functions [155,156]. It indicates how precisely the property value of the output compound is distributed for the input condition. Unlike other metrics, it is not expressed as a specific value, but is usually visualized using a histogram to evaluate the distribution compared to the target value. The smaller the variance, the better the performance.

#### 6.3.2. Pharmacological Indicators

The pharmacological index measures whether the produced compounds have pharmacological effects, through a hypothetical method. Quantitative estimate of drug-likeness (QED), a representative pharmacological index, was introduced by Bickerton et al. [184], and it measures how similar the chemical properties of eight types of drugs are to those of the existing drug groups [185]: molecular weight (MW), lipophilicity (logP), number of hydrogen bond donors (HBD), number of hydrogen bond acceptors (HBA), polar surface area (PSA), number of rotatable bonds (ROTB), number of aromatic rings (AROM), and count of alerts for undesirable substructures. QED was inspired by Lipinski’s rule and was standardized more quantitatively by including the insight. QED is widely used in the de novo drug design; however, usually, researchers evaluate only some of its metrics. Typically, the distributions of the MW and logP are often compared, and HBD and HBA are sometimes used. In particular, the MW and logP are often used to evaluate the control performance of a controllable de novo model [45] and can be considered to produce good performance when the variance is small.

Moreover, synthetic accessibility is measured to enhance the validity of real medicines from a practical industrial perspective. When building a model, synthetic accessibility can be optimized, or it can be filtered by removing compounds that are difficult to synthesize after being randomly generated. Currently, research is underway not only to measure composition difficulty through DL but also to propose suitable synthesis order. If this can be integrated, optimal drugs can be created from the early stages of new drug design considering molecular synthesis.

## 7. Limitation and Future Work

### 7.1. Current Challenges

#### 7.1.1. Data Scarcity and Imbalance

The lack of labeled data is a major limitation to the use of DL-based drug discovery [186]. Data volumes resulting from drug discovery studies are small-scale because it requires expensive experiments and a long time to generate DTI data. For example, the most frequently used benchmark dataset for the DTI prediction is the Yamanishi_2008 dataset [187]. The dataset not only presents data on less than 1000 drugs, but also contains very limited DTI information with an average sparse rate of 3.6% [67].

Besides, the labeled data in drug discovery are extremely imbalanced. Since the HTS technique itself does not presuppose a high frequency of active responses, the HTS data consist of significantly fewer active responses than inactive responses. Consequently, there are often only a few validated drugs available for positive DTIs. In the PubChem Bioassay dataset, an active to inactive ratio of 1:40.92 (a hit rate of 2.385% of the total labeled activity values) indicates that most of the test results are inactive [188].

#### 7.1.2. Absence of Standard Benchmark

In reality, the total number of drugs and proteins tested during the experiment is limited, making it imprecise to guarantee how a specific drug or target protein can work under the same experimental conditions. This problem is prominent in public databases that have accumulated data from the experimental results of numerous researchers around the world. However, big pharmaceutical companies can collect a large amount of data points by analyzing of constant conditions and well-characterized quality [133]. One research group built a model using the company’s private data and the public ChEMBL [189] data and found that the predictive quality of the company model was higher than that of the public data model [115]. This demonstrates that the experimental conditions in the standardized datasets can affect the DNN prediction quality. Therefore, the necessity of data standardization and curation prior to building a predictive model are indispensable. Many public databases, including PubChem [190], ChEMBL, and ExCAPE-DB [191], aimed to standardize and integrate multiple-sourced datasets to facilitate computational drug discovery. However, many DTI prediction models use only a small benchmarking dataset and use the train data and test data from the same source. This shows that many DTI models do not properly validate their generalization performance, demonstrating their inability to predict new DTIs in practical drug development.

### 7.2. Promising Method

#### 7.2.1. Transfer Learning

As mentioned in the previous section, one of the biggest problems in drug discovery using AI is the lack of data. When targeting a specific disease or newly discovered target, the amount of data is so small that it is difficult to train. Moreover, it is difficult to easily apply augmentation to all the data. In such a situation, transfer learning is an excellent alternative [186,192]. Transfer learning, as part of lifelong learning, is inspired by how quickly humans acquire new knowledge from other similar experiences in the past. Transfer learning can improve many problems of insufficient data by fine-tuning a pre-trained model with a large dataset in another or a general field to an actual small-scale dataset [181]. Bonggun et al. [23] imported a molecule representation model learned from the PubChem database and applied it to their DTI model to improve performance. Panagiotis et al. reported that the transfer learning method exhibited improved performance in CHEMBL25 or DRD2 in the de novo study using conditional RNN [182].

**Figure 8:**
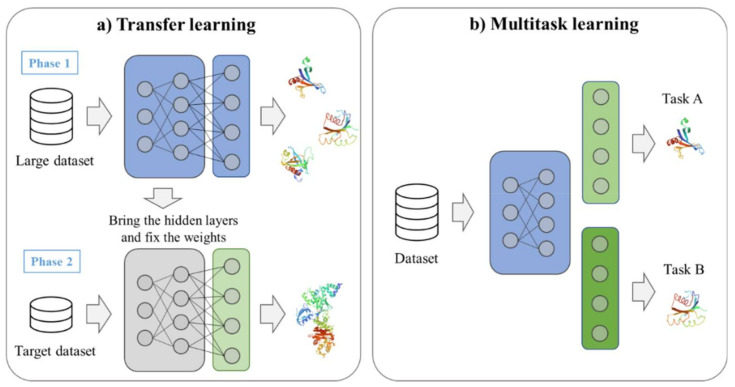
Simple example of transfer learning and multi-task learning. (**a**) Transfer learning (**b**) Multitask learning.

Multi-task learning is also frequently used in drug discovery [186]. If transfer learning is to take the weights of a well-initialized DL model using a large dataset and use it for the target model, multi-task learning trains multiple tasks with many common parts at the same time (Figure 8). With multi-task learning, intrinsic features that are difficult to train with small datasets can be trained using different tasks. Steven et al. showed that using multi-task learning increased the AUC compared to the conventional random forest method or logistic regression method. When using multi-task learning, some datasets exhibited slightly decreased AUC, but for most datasets, AUC increased significantly. In particular, it is noteworthy that the performance of the datasets with a relatively smaller amount of data improved significantly. Using a pre-trained model improves the performance [47]; however, it has the advantage of significantly reducing the training time and computing power from an industrial and practical point of view. Therefore, we recommend using transfer learning for representation learning.

#### 7.2.2. Data Augmentation

There is a method of supplementing the data by incorporating small modifications in the existing data or changing the expression rule, which is called data augmentation. Data augmentation reduces model overfitting and improves the general performance. For data such as the voxel, a common image data augmentation method called geometric transformations can be applied [193]. Alternatively, there is a data augmentation method that adds a small amount of noise that does not affect the performance of the data. Isidro et al. [194] improved the predictive performance of the model by adding Gaussian noise to bioactivities and compound descriptors.

Another popular data augmentation method in drug discovery is randomized SMILES [146,182]. One compound can be written in various SMILES according to the starting point and direction. In the early stage of drug discovery using DL, a canonical SMILES was used for consistent expression; however, in the field of de novo drug design, randomized SMILES is used in a more general way [195]. Josep et al. [182] revealed that the quality of the generative model was better when using randomized SMILES than when using canonical SMILES. Randomized SMILES is mainly used for the de novo drug design [146]; however, Esben [30] showed that randomized SMILES trained more reliably and performed better than the canonical form even when predicting IC50. Unlike de novo, where the number of possible representations of a molecule is important, DTI requires information on the relationship between the ligand and target; therefore, it is not widely used.

#### 7.2.3. Uncertainty and Interpretation

DL is a very powerful tool. It gives us hope that problems that were difficult to address using the classical ML methods can be solved with good performance if high-quality data are supplied abundantly. However, problems arose as the field of application of DL was expanded to a specialized area rather than an easy task. Since the parameters in the model are fixed and the operation process can be known, it is not actually a black box; however, it is treated as a black box because it is difficult for a human to interpret the process of deriving the result [196]. The non-transparency of this interpretation makes it difficult to accurately understand the reasoning process or an obstacle to decision-making. In particular, in areas such as drug discovery or disease diagnosis, where a wrong decision is costly and time consuming, sufficient evidence is needed to accept the result. Therefore, there is a growing need for explainable AI. An explainable AI review paper in the field of drug discovery by Jiménez-Luna et al. describes this well [197].

Although ‘explainable’ is defined in many ways, we will describe only two of the most commonly used concepts [198]. The first is ‘uncertainty estimation’. Uncertainty can be thought of as the opposite of reliability of AI. In the case of the classification model, the weight for each class is output in the last layer, and the class with the highest value is selected using a function such as softmax. However, sometimes, the model outputs completely different results even with very small changes in the weight of the data or hidden layer. From this point of view, uncertainty can be interpreted as a measure of robustness against noise in the training process or model parameters when a certain result is output. Uncertainty leads researchers to make safer and more efficient decisions by estimating risks that will occur during drug development [199]. The second is ‘interpretation’. Interpretation is often used interchangeably with ‘transparency’ depending on the paper [3,198]. Xuhong et al. [64] redefined ‘interpretability’ as follows in their paper: “The model interpretability is the ability (of the model) to explain or to present in understandable terms to a human.” The initial concept of an interpretable DL model was to create class activation maps [200] from the convolution layer of the CNN to visualize the reason for prediction by matching the input result. In the recent drug discovery field, attention-based explainable models dominate. The increased use of attention-based models such as the transformer is also because the performance is better than the other methods at sequential data; however, the reason can be inferred indirectly from attention. Gao et al. [119] created an attention matrix from the results of embedded protein (LSTM) and molecule (GCN). The attention matrix visualized contributing weights of atoms in molecule and residues that affect the DTI, thereby helping researchers to understand the process in a transparent manner and gain new insights. As a solubility prediction method, but not that of DTI, Karpov et al. [77] used a transformer-CNN model from the SMILES data, and Liu et al. [201] used the GCN from a molecule graph to predict the positive or negative contribution of the atoms to solubility. Chen et al. created a model to interpret the atoms contributing to the interaction in the prediction of the DDI [80].

The advantage of interpretability is that it gives the researchers confidence in the results. When the reason for drawing a conclusion is consistent with prior knowledge, the expert can accept the decision with high confidence [3]. It can also provide new inductive inspiration to experts [198]. Finally, it can provide another channel to discover problems when the performance of the DL models is poor.

## Abbreviations

AI	Artificial Intelligence
ADMET	Absorption, distribution, metabolism, excretion, and toxicity
AUC	Area Under the Curve
AUPR	Area Under the Precision–Recall Curve
AE	Autoencoder
CNN	Convolutional Neural Networks
CI	Concordance Index
DDI	Drug–Drug Interaction
MAE	Mean Absolute Error
MCC	Matthews Correlation Coefficient
ML	Machine Learning
MLP	Multi-Layer Perceptron
DL	Deep learning
DTA	Drug–Target Affinity
DTI	Drug–Target Interaction
DTP	Drug–Target Pair
FP	Fingerprint
FPR	False Positive Rate
HBA	Hydrogen Bond Acceptor
HBD	Hydrogen Bond Donor
HTS	High-Throughput Screening
GAN	Generative Adversarial Networks
GCN	Graph Convolutional Networks
GO	Gene Ontology
LINCS	Library of Integrated Network-based Cellular Signatures
LSTM	Long Short-Term Memory
PPI	Protein–Protein Interaction
QSAR	Quantitative Structure-Activity Relationship
RMSE	Root Mean Square Error
RNN	Recurrent Neural Networks
SMILES	Simplified Molecular-Input Line-Entry System
TPR	True Positive Rate
TPSA	Topological Polar Surface Area
VAE	Variational AutoEncoder
VS	Virtual Screening

## Appendix A

**Table A1:** Ligand-based DL methods for DTI prediction.

Reference	Models	Input Drug Type	Datasets	Algorithm Type	Year	Evaluation Metrics
Gao et al. [33]	MLP; Multi-task	Fingerprint (ECFP; FP2; Estate1; Estate2; MACCS; ERG)	PDBbind	Regression	2019	Pearson correlation coefficient (R); RMSE
Wenzel et al. [115]	MLP; Multi-task	Atom pair; pharmacophoric donor–acceptor pairs	ChEMBL	Regression	2019	$R^{2}$
Xie et al. [32]	MLP; LSTM	Fingerprint (MACCS+ECFP)	DrugBank; ChEMBL; PDBbind	Regression	2020	Pearson correlation coefficient (R); RMSE
Hirohara et al. [25]	CNN	SMILES convolution fingerprint	Tox21	Classification	2018	AUC
Matsuzaka et al. [109]	CNN	2D image	Tox21	Classification	2019	AUC; Balanced accuracy; F-score; MCC
Rifaioglu et al. [19]	CNN	2D image	ChEMBL; MUV; DUD-E	Classification	2020	AUC; Accuracy; Precision; Recall; F1-score; MCC
Liu et al. [81]	GCN; Multi-task	3D molecular graph	Amgen’s internal dataset; ChEMBL	Regression	2019	$R^{2}$; Accuracy
Yang et al. [21]	GCN	SMILES	PDBbind; ChEMBL; PubChem Bioassay; MUV; Tox21; ToxCast; SIDER etc.	Classification; Regression	2019	MAE; RMSE; AUC; AUPR
Shang et al. [11]	GCN; Attention-based	Molecular graph	Tox21; HIV; Freesolv; Lipophilicity (MoleculeNet)	Regression	2018	AUC; RMSE

**Table A2:** Structure-based DL methods for DTI prediction.

Reference	Models	Input Drug Type	Input Target Type	Datasets	Algorithm Type	Evaluation Metrics	Year
Wen et al. [37]	MLP	Fingerprint (ECFP)	PSC (protein sequence composition descriptor)	DrugBank	Classification	TPR; TNR; Accuracy; AUC	2017
Chen et al. [57]	MLP	Fingerprint (PubChemFP)	Various protein features *	DrugBank; Yamanishi	Classification	AUC; AUPR	2020
Öztürk et al. [24]	CNN	SMILES	Sequence	Davis; KIBA	Regression	CI; MSE	2018
Shin et al. [23]	CNN; attention	SMILES	Sequence	Davis; KIBA;	Regression	CI; RMSE; $r_{m}^{2}$; AUPR	2019
Zhao et al. [120]	CNN; attention	SMILES	Sequence	Davis; KIBA	Regression	CI; RMSE; $r_{m}^{2}$; AUPR	2019
Gonczarek et al. [202]	CNN	Atom pair	Atom pair	DUD-E; PDBBind	Regression	AUC	2016
Ragoza et al. [203]	CNN	Voxel	Voxel	DUD-E; CSAR	Regression; Classification	AUC	2017
Jiménez et al. [204]	CNN	Voxel	Voxel	PDBbind; CSAR2012	Regression	RMSE; $R^{2}$	2018
Kwon et al. [75]	CNN	Voxel	Voxel	CASF-2016 [205]	Regression	MAE; RMSE	2020
Pu et al. [51]	CNN; multi-classification	Voxel	Voxel	PDB; TOUGH-M1 [206]	Classification	MCC; AUC; Accuracy	2019
Lee et al. [31]	CNN	Fingerprint	Sequence	DrugBank; KEGG; IUPHAR; MATADOR; PubChem Bioassay; KinaseSARfari [189]	Classification	AUC; AUPR; Sensitivity; Specificity; Precision; Accuracy; F1-score	2019
Hasan Mahmud et al. [207]	CNN	SMILES; 193 features by Rcpi	Sequence; 1290 features by PROFEAT	DrugBank; Yamanishi	Regression	AUC; Accuracy; Sensitivity; Precision; F1 score; AUPR	2020
Wang et al. [34]	LSTM	Fingerprint (PubChemFP)	PSSM; Legendre Moment [208]	DrugBank; Yamanishi; KEGG; SuperTarget	Classification	AUC; Accuracy; TPR; Specificity; Precision; MCC	2020
Tsubaki et al. [209]	GNN; CNN; attention	Fingerprint (PubChemFP)	Sequence; Pfam domain	DUD-E; DrugBank; MATADOR	Classification	AUC; Precision; Recall	2019
Torng and Altman [118]	GCN	Molecular graph	Molecular graph	DUD-E; MUV	Classification	AUC	2019
Feng et al. [8]	GCN	Fingerprint (ECFP); 3D molecular graph	PSC (protein sequence composition descriptor)	Davis; Metz; KIBA; ToxCast	Regression	$R^{2}$	2019
Jiang et al. [210]	GNN	3D molecular graph	3D molecular graph	KIBA; Davis	Regression	$r_{m}^{2}$; CI; MSE; Pearson correlation coefficient; Accuracy	2020

* CTD; CT; Pseudo AAC; Pseudo PSSM; NMBroto; Structure feature from SPIDER.

**Table A3:** Relationship-based DL methods for DTI prediction.

Reference	Models	Relationship Data Type	Datasets	Algorithm Type	Evaluation Metrics	Year
Xie et al. [71]	MLP	LINCS signature	DrugBank; CTD; DGIdb; STITCH	Regression	Accuracy; F-score; TPR	2018
Lee and Kim [63]	MLP; node2vec	LINCS signature; PPI (Protein-protein interaction); Pathway	LINCS; ChEMBL; TTD; MATADOR; KEGG; IUPHAR; PharmGKB; KiDB	Classification	AUC; Precision	2019
Gao et al. [119]	CNN; LSTM	LINCS signature; GO term	BindingDB	Regression	Accuracy; AUC; AUPR	2018
Shao and Zhang [147]	CNN; GCN	LINCS signature	LINCS; DrugBank	Classification	Accuracy; AUC	2020
Thafar et al. [67]	node2vec	Drug similarity (structure, side effects); Target similarity (sequence, GO); PPI	Yamanishi; KEGG; BRENDA; SuperTarget; DrugBank; BioGRID; SIDER	Classification	AUPR; AUC	2020
Zong et al. [13]	DeepWalk [130]	Drug-target association; Drug-disease association; Disease-target association	DrugBank; Human diseasome [211]	Classification	AUC	2017
Mongia and Majumdar [212]	Multi-graph deep matrix factorization	Drug similarity (structure); Target similarity (sequence)	Yamanishi; KEGG; BRENDA; SuperTarget; DrugBank	Classification	AUPR; AUC	2020
Wang et al. [59]	AE	Drug similarity (structure, side effects); Target similarity (sequence, GO); PPI	Yamanishi; KEGG; BRENDA; SuperTarget; DrugBank; SIDER	Classification	AUPR; AUC	2020
Zhao et al. [68]	CNN; AE	Drug similarity (structure); Target similarity (sequence); PPI	DrugBank; STRING	Classification	Accuracy; AUPR; AUC	2020
Peng et al. [97]	CNN; AE	Drug-target association; Drug-disease association; Disease-target association; Drug similarity (structure, side effects); Target similarity (sequence, GO); PPI	DrugBank; Human Protein Reference Database [2009]; CTD; SIDER;	Classification	AUPR; AUC	2020
Zhong et al. [213]	GCN	LINCS signature; PPI	ChEMBL; LINCS; STRING	Classification	Accuracy; F-score; AUPR; Precision; Recall; AUC	2020

**Table A4:** Benchmark datasets for DTIs.

Dataset	No. of DTIs	No. of Target	No. of Drug	Year	Availability *
PharmGKB [214]	777	1030	4078	2020	https://www.pharmgkb.org/downloads
Yamanishi [215]	5127	989	932	2008	https://members.cbio.mines-paristech.fr/~yyamanishi/pharmaco/
DrugBank [216]	6566	4844	18,734	2020	https://go.drugbank.com/releases/latest
IUPHAR [217]	6605	1577	14,981	2020	https://www.guidetopharmacology.org/download.jsp
SuperTarget/MATADOR [218]	8936	1799	719	2008	http://matador.embl.de/
DGIdb [219]	11,137	3820	58,555	2020	https://www.dgidb.org/downloads
CTD [220]	17,814	46,364	2,521,525	2020	http://ctdbase.org/downloads/
TTD [221]	18,351	1814	29,388	2020	http://db.idrblab.net/ttd/full-data-download
Davis [113]	27,621	379	68	2011	https://tdcommons.ai/multi_pred_tasks/dti/#davis
Tox21	77,946	12	7831	2014	https://deepchemdata.s3-us-west-1.amazonaws.com/datasets/tox21.csv.gz
Metz [129]	103,920	172	3858	2011	https://www.nature.com/articles/nchembio.530
KIBA [222]	118,036	229	2068	2014	https://tdcommons.ai/multi_pred_tasks/dti/#kiba
MUV [168]	249,886	17	93,087	2009	https://deepchemdata.s3-us-west-1.amazonaws.com/datasets/muv.csv.gz
BindingDB [223]	456,248	3716	747,066	2020	https://www.bindingdb.org/bind/
ToxCast [224]	530,605	335	7675	2007	https://www.epa.gov/chemical-research
ExCAPE-DB [191]	582,724	1667	1,361,473	2017	https://solr.ideaconsult.net/search/excape/
DUD-E [166]	1,167,186	102	22,886	2012	http://dude.docking.org/

* Site accessed date: 14 September 2021.
